# Get Smart About Antibiotics Week — November 17–23, 2014

**Published:** 2014-11-14

Each year, an estimated 2 million persons in the United States are infected with antibiotic-resistant bacteria, and approximately 23,000 die as a result (1). The rise of antibiotic resistance represents a serious threat to human and animal health, national security, and economies worldwide.

The use of antibiotics is the single most important factor leading to antibiotic resistance around the world. In September, the White House issued an executive order and announced the National Strategy to Combat Antibiotic-Resistant Bacteria.* These actions provide goals and direction to help the nation contain the spread of resistant bacterial strains, manage existing antibiotics to preserve their effectiveness, and help ensure a steady pipeline of new, effective antibiotics and diagnostics.

CDC will leverage its expertise and build on core strengths to address the threat of antibiotic resistance to slow the development of resistant bacteria and prevent the spread of resistant infections by 1) strengthening national surveillance efforts to track resistant bacteria, 2) advancing development and use of rapid and innovative diagnostic tests for identification and characterization of resistant bacteria, and 3) improving international collaboration and capacities for antibiotic resistance prevention, surveillance, control, and antibiotic research and development.

This year, Get Smart About Antibiotics Week is being observed during November 17–23, 2014. This is an annual observance to raise awareness of the threat of antibiotic resistance and the importance of appropriate prescribing and use. The observance is a key component of CDC’s efforts to improve antibiotic stewardship in communities, in health care facilities, and on farms in collaboration with state-based programs and others. Get Smart About Antibiotics Week coincides with many global antibiotic resistance observances, including those in Europe, Australia, and Canada. Information on scheduled activities and how to get involved in combating antibiotic resistance is available at http://www.cdc.gov/getsmart/week.

## Footnotes

* Additional information available at http://www.whitehouse.gov/sites/default/files/docs/carb_national_strategy.pdf.
